# Differential Etching of Rays at Wood Surfaces Exposed to an Oxygen Glow Discharge Plasma

**DOI:** 10.3390/ma17020521

**Published:** 2024-01-22

**Authors:** Kenneth J. Cheng, Weicong Ma, Philip D. Evans

**Affiliations:** Department of Wood Science, University of British Columbia, Vancouver, BC V6T 1Z4, Canada; kyeser@mail.ubc.ca (K.J.C.); vickiema@student.ubc.ca (W.M.)

**Keywords:** plasma, glow-discharge, basswood, etching, microstructure, rays

## Abstract

Basswood samples were exposed to oxygen glow-discharge plasmas for 30 min, and etching of radial and tangential longitudinal surfaces was measured. It was hypothesized that there would be a positive correlation between etching and plasma energy, and differential etching of wood surfaces because of variation in the microstructure and chemical composition of different woody tissues. Etching at the surface of basswood samples was examined using profilometry. Light and scanning electron microscopy were used to examine the microstructure of samples exposed to plasma. There was a large effect of plasma energy on etching of basswood surfaces, and radial surfaces were etched to a greater extent than tangential surfaces. However, rays at radial surfaces were more resistant to etching than fibers, resulting in greater variation in the etching of radial versus tangential surfaces. The same phenomenon occurred at radial surfaces of balsa wood, jelutong and New Zealand white pine subjected to plasma etching. The possible reasons for the greater resistance of rays to plasma etching are explored, and it is suggested that such differential etching of wood surfaces may impose a limitation on the use of plasma to precisely etch functional patterns at wood surfaces (raised pillars, grooves), as has been done with other materials.

## 1. Introduction

Plasma is an ‘energetic ionized gas consisting of atoms, molecules, ions, free radicals, electrons and metastable species’ [1]. The excited species in plasmas can chemically and physically modify the surface of materials via one or a combination of the following mechanisms: surface cleaning; degradation and etching; cross-linking; polymerization; grafting and ion implantation. Plasma has been used to increase the wettability of wood to improve its suitability as an adherend for glues and coatings [2,3,4], remove unwanted microbiological stains from wood [5,6], and create hydrophobic coatings at wood surfaces [7]. These practical applications of plasma for wood surface modification mirror previous research on the plasma modification of other materials [8,9,10]. In addition, plasma treatments have been used for VOC abatement [11,12], odor removal in waste air [13,14], advanced waste water treatments [15,16], pretreatment of drinking water [17,18,19] and disinfection of air, water and solid materials [20,21,22]. However, the most important commercial application of plasma is for the etching of surfaces. For example, plasma etching of microstructural features on silicon has been critical to the development of integrated circuits (chips), micromachines (MEMS), and optical devices, ‘without which the world would be a very different place’ [1,23,24]. Plasma etching of silicon involves placing a mask on the substrate and then etching the assembly with a plasma that etches the substrate while sparing the mask [1]. A variety of different microstructures can be created by plasma etching of silicon, including trenches, holes, cones, pillars and wires [25,26,27]. 

Plasma etching to create specific microstructures at wood surfaces has received little attention but is potentially interesting because such features could alter important wood properties such as surface wettability [28], permeability [29] and distribution of strains [30]. The creation of specific microstructures in materials requires a high degree of etch uniformity both within and between samples [1]. Such uniformity has been achieved with silicon [1]. However, it seems unlikely that a high degree of etch uniformity can be achieved with wood because of spatial variation in its microstructure and chemical composition between and within wood species. Both have been shown to influence the plasma etching of wood. For example, thicker-walled tracheids in yellow cedar (*Callitropsis nootkatensis* D. Don) are more prominent than thinner-walled tracheids following plasma etching [31], and lignin-rich areas of cell walls such as the middle lamella are more resistant to etching than areas rich in holocellulose [32,33,34]. 

The main purpose of this work is to (1) test the hypothesis that there will be differential etching of basswood surfaces because of variation in the microstructure and chemical composition of different woody tissues; (2) examine the effect of plasma energy on etching of basswood. Results showed a large effect of plasma energy on etching of basswood surfaces and large within-sample variation in etching of both radial and tangential longitudinal surfaces. Radial surfaces were etched to a greater extent than tangential surfaces, but rays at radial surfaces were more resistant to etching than fibers, resulting in greater variation in the etching of radial surfaces compared to tangential surfaces. Differential etching of rays also occurred at radial surfaces of balsa wood, jelutong and New Zealand white pine subjected to plasma etching. The possible reasons for the greater resistance of rays to plasma etching and the practical implications of the differential etching of wood surfaces are discussed.

## 2. Materials and Methods

### 2.1. Wood Samples

Basswood (*Tilia americana* L.) and jelutong (*Dyera costulata* (Miq.) Hook.f) boards of different sizes were purchased from a retailer of lumber (PJ White Hardwoods, Vancouver). Balsa wood (*Ochroma pyramidale* (Cav. ex Lam.) Urb.) and New Zealand white pine (*Dacrycarpus dacrydioides* (A. Rich.) de Laub.) specimens were retrieved from wood collections at The Australian National University and University of British Columbia, respectively. Wood specimens were sawn with a band saw (Meber SR-500, Carpi, Italy) to produce samples measuring 62 mm (long) × 18 mm (wide) × 5 mm (thick) with radial or tangential surfaces facing uppermost. Samples were hand-sanded down to 3.8 mm thickness using a sequential series of abrasive papers: 100, 150, and 500 grit (3 M OG32, Home Depot, Vancouver, BC, Canada). 

### 2.2. Plasma Treatment

Prior to plasma treatment, samples were placed under vacuum (−95 kPa) for 48 h to remove air from the wood. Samples were secured in a stainless steel bracket with a basal area of 114 × 70 mm. The edges of each sample were held down by two stainless steel strips measuring 106 × 19 × 0.58 mm and two 37 mm long stainless-steel Philips flat head (8.7 mm) machine screws. These stainless steel strips masked the edges of wood samples from plasma so the masked surface could be compared with plasma-treated surfaces. The bracket containing wood samples was secured to a holding arm within the plasma reactor and was located 100 mm from the plasma source. A purpose-built plasma reactor (Flarion Series Model FLR600-M, Plasmionique Inc., Quebec, QC, Canada) was used to generate oxygen plasma. The plasma reactor used an inductively coupled plasma (ICP) source (spiral-wound planar antenna) on an 8 inch (203.2 mm) top port powered by an RF generator with an automatic matching circuit. The planar antenna was water-cooled and located outside of the main chamber to prevent it from contaminating the plasma. The planar antenna consisted of silver-plated 6.35 mm copper tubing wound in a 16.2 cm diameter spiral. To operate the plasma reactor, power was connected to it, and it was ‘warmed up’ for at least 20 min. Compressed air was fed into the mass flow controllers (Aalborg GFC17, Aalborg Instruments and Controls Inc., Orangeburg, NY, USA) (these controlled the gas flowing into the chamber) at 413.7 to 551.6 kPa by using an external 90 liter oil-free air compressor (Kobalt #0322041, Lowes, Mooresville, NC, USA). Water at a minimum temperature of 15 °C and 206.8 kPa was used to cool the plasma source antenna. The sample holder was loaded into the main chamber’s holder, and the chamber door was closed and tightened. An external tank containing oxygen gas was opened up, and the pressure was set at 103.4 kPa. A turbomolecular pumping unit (Pfeiffer HiCube80 Eco, Boisbriand, QC, Canada) evacuated the chamber down to 10^−6^ Torr (1.33 × 10^−7^ kPa), which was measured using Pirani (InstruTech CVG101, Longmont, CO, USA) and Bayard-Alpert (InstruTech IGM402) gauges. The gate valve to the main chamber and its two-stage rotary vane vacuum pump (Vacuum Research Model 600-21, Vacuum Research Corp., Pittsburgh, PA, USA) was opened, and the pump was turned on. Once the main chamber pressure reached ~10^−1^ Torr 1.33 × 10^−2^ kPa, which was monitored by the Pirani gauge and a capacitance manometer (MKS Baratron^®^ 626 B, MKS Instruments Inc., Andover, MA, USA), the gate valve connecting the main chamber and a secondary RGA (residual gas analyzer) chamber was opened and the pressure of the two chambers equalized. Then, the RGA pump was turned off, and the chamber between the main and RGA chamber was closed again, with the main pump controlling the pressure. Once the capacitance manometer read 110 mTorr, an oxygen gas line was opened. A gas flow rate of 0.017148 g/min was used, which increased the chamber pressure. After the main pump evacuated the chamber down to 110 mTorr, the plasma power level and treatment time were set. Different plasma powers (200, 250, 300, 400, 500 W) were used to modify the different surfaces of basswood samples. The second experiment involving the other wood species used 300 W plasma power and a treatment time of 30 min. Controls were kept in an oxygen-rich environment at 0.017148 g/min and held at 110 mTorr for 30 min. Opening the oxygen gas tank maintained the vacuum at the required level. After treatment, the system was shut down, and samples were removed from the chamber.

### 2.3. Macroscopic Imaging

Plasma-treated basswood samples and controls were scanned using a deck-top scanner (Microtek Scanmaker i800, Microtek Int. Inc, Hsinchu, Taiwan) using the following parameters: 600 dpi resolution; True Colours; 100% scale. The morphological changes at the radial surfaces of balsa wood, basswood, jelutong and New Zealand white pine were assessed using a digital microscope (AD206, Shenzhen Andonstar Technology Co. Ltd., Shenzhen, Guangdong, China) connected to a high-resolution (1080 P) camera. A Canon 5D Mark II camera (Canon Inc., Tokyo, Japan) equipped with a macro lens (Canon MP-E 65 mm f/2.8 1-5x) was attached to a Polaroid MP-4 Land Camera Stage (Polaroid Corp., Minnetonka, MN, USA) and used to capture images of the etching (erosion) of plasma-treated basswood samples.

### 2.4. Scanning Electron Microscopy (SEM)

A razorblade (#9 Steelback Carbon Steel Blade, Stanley, New Britain, CT, USA) was used to cut small specimens measuring 2–5 × 5–10 mm from plasma-treated samples and the controls [4]. Specimens were mounted on 12 mm diameter aluminum SEM stubs (2–4 specimens per stub) using double-sided sticky tabs (#76762-0, Electron Microscopy Services, Hatfield, PA, USA). Stubs were coated with 30 nm layer of gold/vanadium alloy using a sputter coater (208 HR High Resolution Sputter Coater, Cressington Scientific Instruments, Watford, UK) and examined using a field emission scanning electron microscope (Zeiss Ultraplus, Jena, Germany) with an accelerating voltage of 5 kV and working distances of 14.1 to 15.0 mm. SEM images were saved as TIFF files.

### 2.5. Confocal Profilometry

Plasma-treated basswood samples and the control were scanned using a confocal profilometer (Altisurf 500*^®^*, Altimet, Marin, France). Image analysis software (Altimet Premium, v. 6.2.) was used to obtain the depth of etching and to provide topographical images of the plasma-treated samples [33]. To quantify depth of etching, a 3 × 3 mm area containing the masked and unmasked parts of the plasma-treated surface was scanned with a 3 mm probe at a resolution of 301 × 301 points and a spacing of 10 µm. Three profiles were extracted from every measured surface, and these profiles were used to determine the depth of the etched areas. For each profile, six measurements were obtained, and the mean height difference was obtained from the heights of the masked and unmasked areas. To accurately measure etching depth, a waviness profile was used to remove checks and prevent them from being included in the etching measurements [35]. Analysis of variance was used to examine the effects of plasma power on the depth of etching of samples. Statistical computation was performed using Genstat (v. 21, VSNi, Hemel Hempstead, UK).

### 2.6. Chemical Analysis

Two quarter-sawn wood blocks measuring 60 mm (tangential) × 20 mm (radial) × 100 mm (longitudinal) were cut from separate basswood boards. Blocks were immersed in distilled water for 7 days. Veneers 60 to 80 μm thick were cut from the radial face of the small blocks using a microtome (Spencer Lens, Buffalo, New York, NY, USA) with a blade holder (Feather No. 160, Feather Safety Razor Co. Ltd., Osaka, Japan) and disposable microtome blades (Feather Type S35, Feather Safety Razor Co. Ltd., Osaka, Japan). A single-edged razor blade (#9 Steelback Carbon Steel Blade, Stanley, New Britain, CT, USA) was used to cut ray and non-ray tissues from these veneers and the resulting tissue fractions were ground in a low-loss rotary mill (A10, Janke and Kunkel, IKA Works Inc. Wilmington, NC, USA) to pass a 40-mesh screen and extracted with cold water and acetone [36]. The following analyses were made on duplicate 200 mg samples: (a) acid-insoluble lignin; (b) acid-soluble lignin [37]. The acid-insoluble, acid soluble and total lignin contents of ray tissue (*w*/*w*) were 22.0%, 1.9%, and 23.9%, respectively. Comparable figures for non-ray tissues were 20.7%, 1.6%, and 22.3%, respectively.

## 3. Results

### 3.1. Erosion Measurements and Confocal Profilometry Images of Plasma Etched Basswood Surfaces

**Figure 1:**
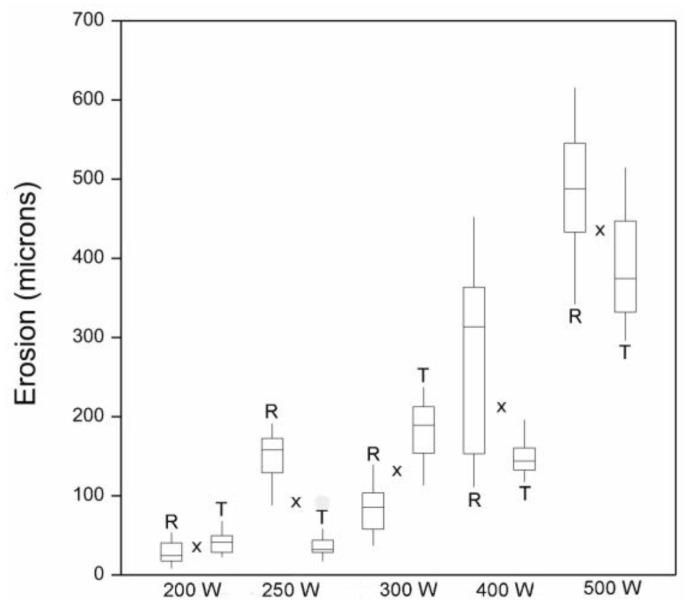
Erosion of basswood surfaces exposed to a glow discharge plasma with different energy levels (W) for 30 min. R = radial longitudinal and T = tangential longitudinal. Boxes represent interquartile range. Line within each box is the median, and whiskers represent data range. X = mean.

There was a positive curvilinear relationship between plasma energy (W) and erosion of basswood samples, and the effect of plasma energy on erosion was statistically significant (*p* < 0.001) (Figure 1). 

**Figure 2:**
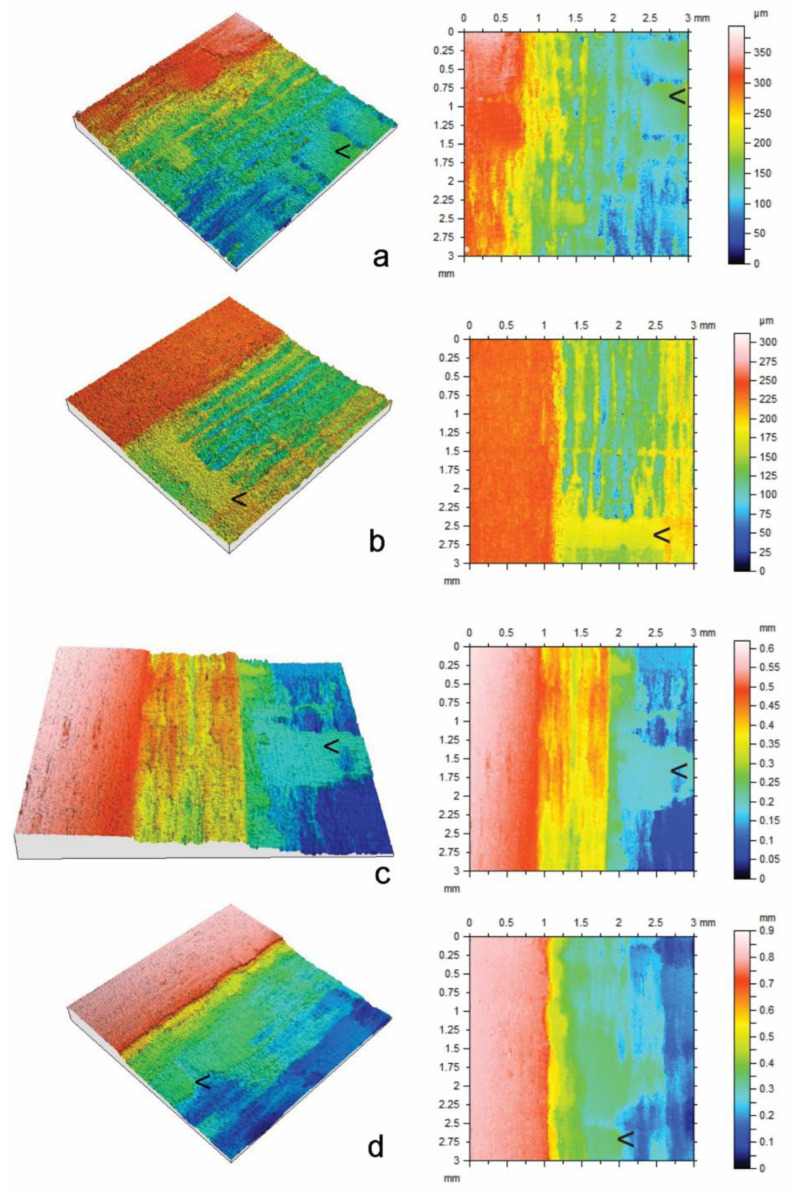
Confocal profilometry height maps of radial longitudinal basswood surfaces exposed to glow discharge plasma with different energy levels (W) for 30 min: (**a**) 250 W; (**b**) 300 W; (**c**) 400 W; (**d**) 500 W. Arrows (<) indicate areas that were less susceptible to etching.

**Figure 3:**
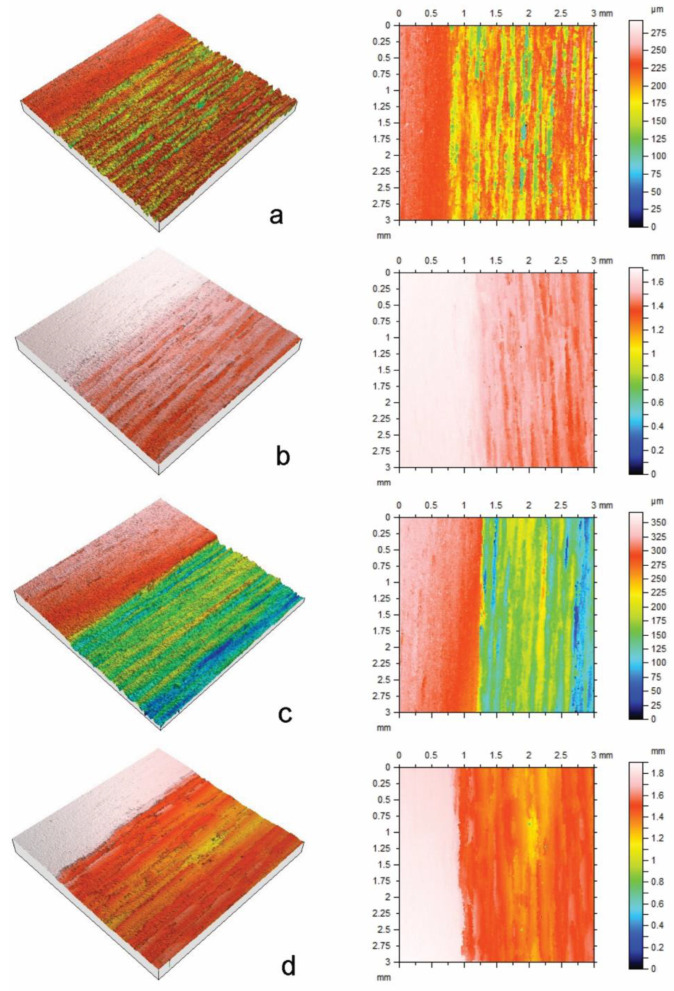
Confocal profilometry height maps of tangential longitudinal basswood surfaces exposed to glow discharge plasma with different energy levels (W) for 30 min: (**a**) 250 W; (**b**) 300 W; (**c**) 400 W; (**d**) 500 W.

In comparison to the effects of plasma energy on erosion, the effects of surface type (radial or tangential longitudinal) and the interaction of plasma energy and surface type on erosion were smaller, although statistically significant (*p* < 0.05). Erosion of radial surfaces exposed to high-energy plasma (400 or 500 W) was greater than that of similarly exposed tangential surfaces. In addition, there was greater etch non-uniformity of radial surfaces compared to tangential surfaces, as can be seen by comparing paired box plots (radial v tangential) at different energy levels in Figure 1. The greater variability of etching at radial compared to tangential surfaces can also be seen in confocal profilometry height maps of samples exposed to plasma (Figure 2 and Figure 3). 

The individual confocal profilometry height maps in Figure 2 and Figure 3 for radial and tangential longitudinal basswood surfaces, respectively, show the uneroded masked regions on the left and etched regions to the right. The height maps are adjusted to include erosion minima and maxima. Within the etched regions of radial longitudinal surfaces, there are areas that are less severely etched than others, including thin vertically aligned bands, which are noticeable in Figure 2b, and broad bands of tissue that are arrowed within each height map. The former is also present in etched tangential longitudinal surfaces (Figure 3), but broad bands of tissue that are less resistant to etching are absent from tangential longitudinal surfaces. 

### 3.2. Microstructure of Plasma Etched Basswood Surfaces

**Figure 4:**
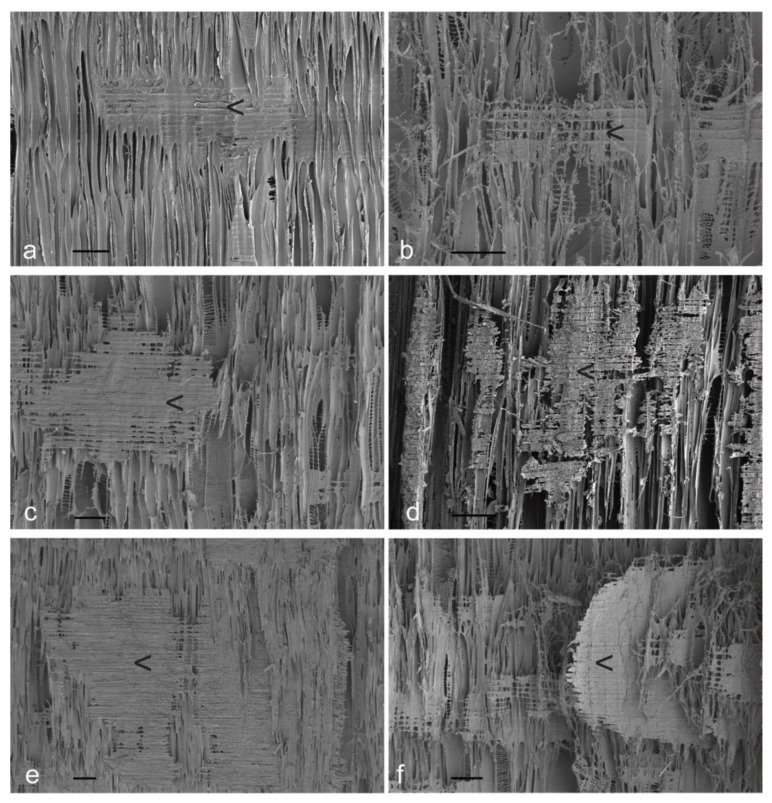
Scanning electron photomicrographs of radial longitudinal basswood surfaces exposed to different plasma energy levels (W) for 30 min: (**a**) Unexposed control; (**b**) 200 W; (**c**) 300 W; (**d**) 300 W; (**e**) 400 W; (**f**) 500 W. Scale bars at the bottom left of each image = 100 μm.

Scanning electron photomicrographs of radial longitudinal basswood surfaces exposed to different plasma energy levels for 30 min are shown in Figure 4. It is clear from SEM images in Figure 4 that the broad bands of tissue that confocal profilometry revealed as being resistant to etching are rays (arrowed [<] in each image, below).

**Figure 5:**
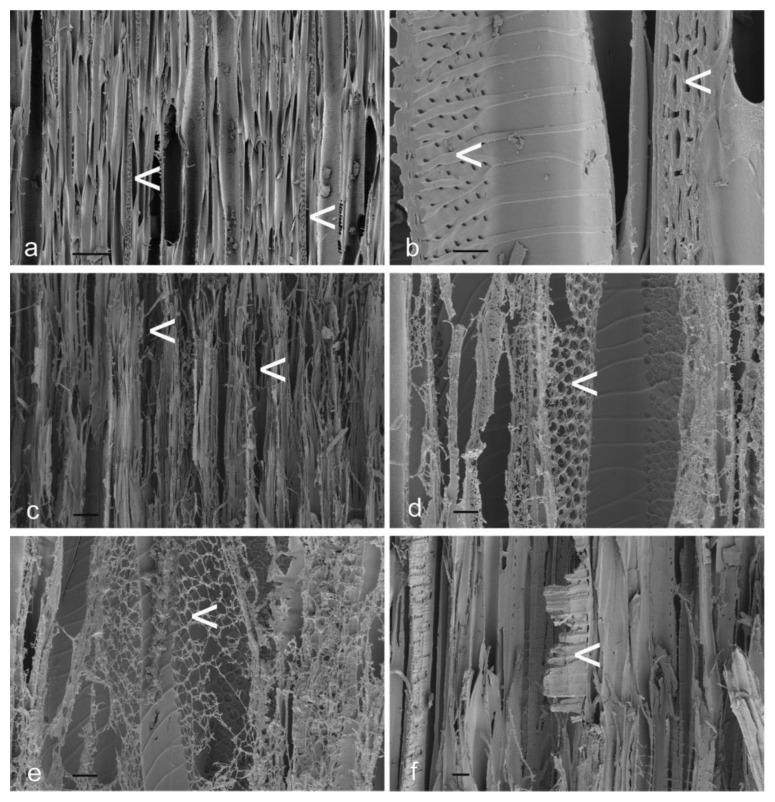
Scanning electron photomicrographs of tangential longitudinal basswood surfaces exposed to different plasma energy levels (W) for 30 min: (**a**) Unexposed control; (**b**) 200 W; (**c**) 300 W; (**d**) 300 W; (**e**) 400 W; (**f**) 500 W. Scale bars at the bottom left of each image = 100 μm.

In addition to the greater resistance of rays to plasma etching, SEM images confirm previous observations that bordered pits on vessel walls are rapidly etched, whereas helical thickening on vessel walls is retained for longer during plasma etching than intervening vessel wall material (Figure 4b,c) [31]. Erosion of vessels appears to create vertically aligned voids that are apparent in confocal profilometry images of plasma-etched radial longitudinal surfaces (Figure 2). SEM images of tangential longitudinal surfaces exposed to different plasma energy levels for 30 min show more clearly how vertically orientated voids are created (Figure 5). Untreated tangential longitudinal surfaces display cut ends of rays, pit fields and thickening on vessel walls (arrowed [<] in Figure 5a,b). After plasma treatment, voids develop at tangential surfaces (arrowed in Figure 5c). These voids appear to be created by erosion of pit fields on vessel walls (arrowed in Figure 5d,e). The cut ends of rays project from plasma-etched tangential longitudinal surfaces (arrowed in Figure 5f).

### 3.3. Macroscopic Changes at Plasma Etched Surfaces

**Figure 6:**
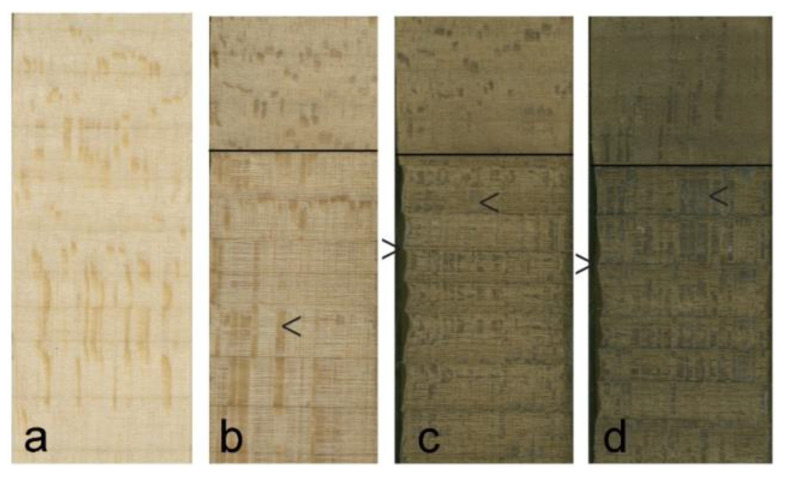
Images of radial longitudinal basswood surfaces exposed to different plasma energy levels for 30 min: (**a**) Unexposed control; (**b**) 300 W; (**c**) 400 W; (**d**) 500 W. Note that rays are either longer or wider in the region below the black line that was fully exposed to plasma.

The retention of rays at plasma etched surfaces that is apparent microscopically could also be seen with the naked eye. Figure 6 shows scans of radial longitudinal basswood surfaces exposed to different plasma energy levels for 30 min. The upper part of each specimen (above the horizontal black line) was covered by a mask and was not directly exposed to plasma (Figure 6), although with increasing plasma energy, there is darkening of the masked area, possibly due to thermal oxidation of wood. The lower part of each specimen was exposed to plasma. Rays are much more prominent in the etched part of the radial longitudinal specimens than in the upper (masked) part (arrowed [< or >] in Figure 6).

**Figure 7:**
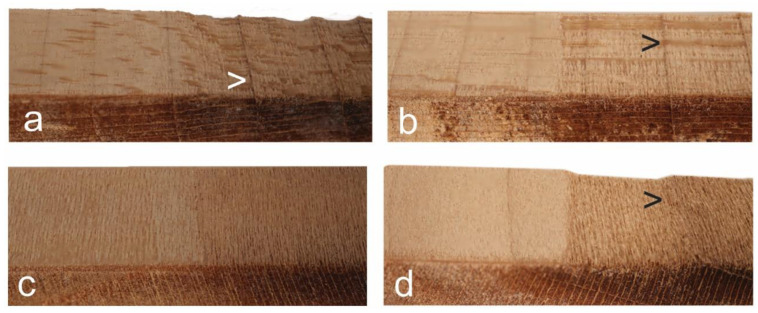
Macroscopic photography images of basswood samples exposed to plasma for 30 min. Left-hand side of each sample was masked and the right-hand side was exposed to plasma: (**a**) Radial longitudinal surface exposed to 250 W plasma. Note the latewood ridges protruding from the surface of the specimen (arrowed); (**b**) Radial longitudinal surface exposed to 300 W plasma. Note the latewood ridges at the surface of the specimen and the prominent rays (arrowed); (**c**) Tangential longitudinal surface exposed to 250 W plasma. Note the even surface; (**d**) Tangential longitudinal surface exposed to 300 W plasma. Note the latewood ridge at the surface of the specimen (arrowed), where a band of latewood intersects the surface at an oblique angle.

In addition, it is possible to see that the band of dense latewood fibers that delimit growth rings in basswood [38] is less eroded than earlywood fibers following plasma treatment (Figure 6). Such an effect is more pronounced at radial surfaces where bands of latewood fibers are aligned at 90° to radial surfaces (arrowed [>] in Figure 7a,b). It also occurs at tangential surfaces if latewood bands are not completely parallel to the surface exposed to plasma (arrowed [>] in Figure 7d). 

**Figure 8:**
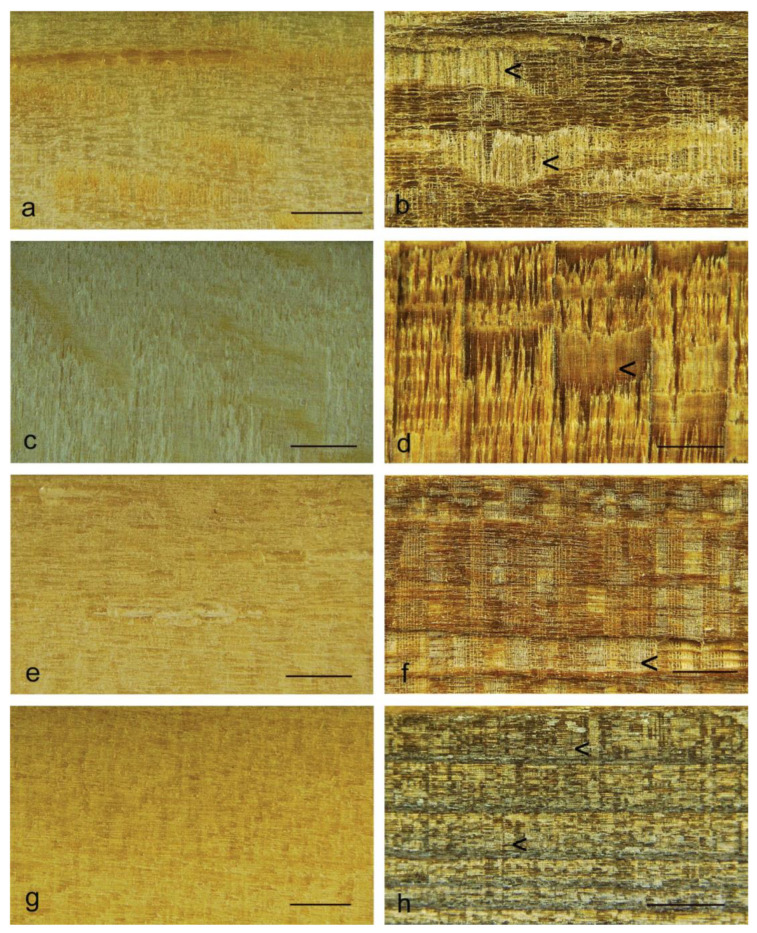
Images of radial longitudinal surfaces before and after exposure to plasma (300 W) for 30 min: (**a**) Unexposed balsa wood control; (**b**) Plasma-treated balsa wood; (**c**) Unexposed basswood control; (**d**) Plasma-treated basswood; (**e**) Unexposed jelutong control; (**f**) Plasma-treated jelutong; (**g**) Unexposed New Zealand white pine control; (**h**) Plasma-treated New Zealand white pine. Scale bars at the bottom right of each image = 1 mm.

The greater resistance of rays in basswood to plasma etching can also be observed in low-power microscopy images (Figure 8c,d). Figure 8 also contains images of radial surfaces of three additional wood species (balsa wood, jelutong and New Zealand white pine) subjected to plasma etching. Rays in these species, in addition to those in basswood, also appear to be more resistant to plasma etching than other tissue types (arrowed [<] in images in Figure 8). 

**Figure 9:**
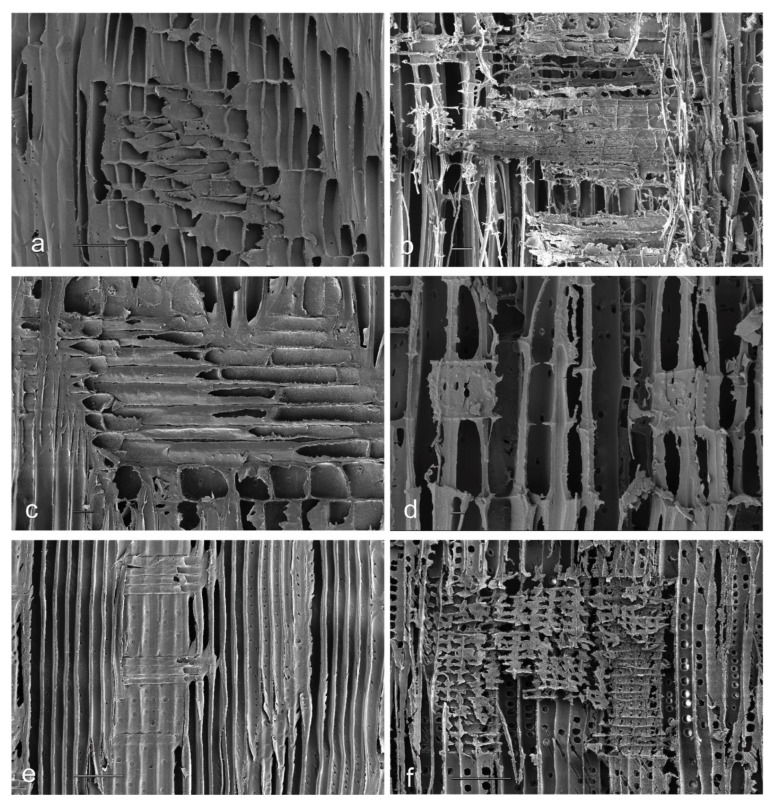
Scanning electron photomicrographs of radial longitudinal wood surfaces exposed to plasma (300 W) for 30 min, and the untreated controls: (**a**) Unexposed balsa wood control; (**b**) Plasma-treated balsa wood; (**c**) Unexposed jelutong control; (**d**) Plasma-treated jelutong; (**e**) Unexposed New Zealand white pine control; (**f**) Plasma-treated New Zealand white pine. Scale bar = 10 μm.

SEM photomicrographs confirmed that rays in balsa wood, jelutong and New Zealand white pine were more resistant to plasma etching than other tissue types (Figure 9). 

## 4. Discussion

In the introduction to this paper, it was hypothesized that there would be differential etching of basswood surfaces by plasma. The results above support this hypothesis because plasma-etched basswood surfaces varied in topography. Variation in topography occurred at both microscopic and macroscopic length scales. Microscopic variation in topography of both radial and tangential surfaces occurred because etching of bordered pit fields on vessel walls led to more complete erosion of vessels compared to fibers, which created vertically aligned voids at surfaces exposed to high-energy plasma. Macroscopic variation in topography occurred because of differential etching of latewood and earlywood tissues, resulting in bands of latewood projecting from etched surfaces. Such an effect was pronounced at radial longitudinal surfaces and only occurred at tangential longitudinal surfaces if growth rings were not parallel to the surface. The differential etching of latewood and earlywood tissue in basswood accords with previous research showing that reductions in dimensions of thick-walled softwood tracheids following plasma treatment are less than those of thinner-walled tracheids [31]. Similarly, and in accordance with our findings, ‘etch rates for porous silicon are generally higher than for solid silicon due to the inherent smaller mass density of the latter’ [39].

The greater resistance of rays to plasma etching in comparison to longitudinally oriented tissues also contributed to the greater variation in topography of radial longitudinal surfaces in comparison to tangential longitudinal surfaces. The persistence of ray tissues at radial longitudinal basswood surfaces subjected to plasma etching could be seen with the naked eye and also microscopically. Observations of similarly treated radial longitudinal surfaces of two hardwoods (balsa wood and jelutong) and a softwood (New Zealand white pine) confirmed that rays were resistant to plasma etching. Ray cells in basswood were thick-walled, and the density of rays is greater than that of surrounding tissues, resulting in a positive correlation between ray volume and wood density [40,41]. Hence, it is possible that the retention of rays at plasma-treated radial surfaces is related to their relatively high density, in accordance with the greater resistance of latewood to plasma etching compared to earlywood. The lignin content of rays in basswood was greater than that of non-ray tissues (23.9% versus 22.3%). Lignin is more resistant to plasma etching than hemicellulose and cellulose [33], and therefore, the higher lignin content of rays may also explain why they persisted at plasma-etched surfaces. 

The persistence of rays at radial surfaces exposed to high-energy plasma has not been reported previously, thus our findings cannot be compared with others in the literature. However, plasma treatment shares some similarities with gamma irradiation as both irradiate wood with high-energy particles, and the effects of gamma radiation on wood resemble those resulting from prolonged plasma treatment [42,43]. For example, both plasma treatment and gamma irradiation preferentially degrade hemicelluloses and celluloses, and as a result, lignin-rich regions of wood cell walls, such as the middle lamella and S3 layer, become more prominent following the treatments [32,33,42,43]. One study of the ultrastructure of gamma-irradiated Douglas fir (*Pseudotsuga menziesii* (Mirbel) Franco) and tulip wood or yellow poplar (*Liriodendron tulipifera* L.) noted that the cell walls of ray parenchyma were more resistant to gamma irradiation than tracheid walls [42]. The greater gamma radiation resistance of ray cells was thought to be related to their high lignin content [42]. The rays in basswood were more resistant to plasma etching than some non-ray tissues, and their lignin content was higher. Therefore, our findings are in accordance with previous research on the relative resistance of ray parenchyma and prosenchyma to degradation by gamma radiation [42].

As mentioned in the introduction to this paper, plasma etching of wood was of interest due in part to its potential to selectively etch surfaces to create patterned wood surfaces with improved functionality. Uniformity or good lateral resolution of plasma etching is needed to create reproducible microstructures in materials such as silicon wafers [23]. Such etch uniformity is more easily achieved with solid materials such as silicon and metals [23,44], but it has also been achieved with porous silicon [45]. Wood is a highly porous material depending on its density and there is great variation in its microstructure [38]. Results here showed that the uniformity of etching of tangential basswood surfaces was better than that of radial surfaces, and hence, the former might be more suitable for microstructural modification by plasma etching. However, microscopic variation in surface topography was present at both surfaces because of variation in the etching of vessels and fibres. Such etch non-uniformity could be overcome by etching very dense hardwoods with a high proportion of thick-walled fibers and small vessels. Accordingly, research has been undertaken to examine the ability of plasma to etch the very dense wood species lignum vitae (*Guaiacum officinale* L.), but this was unsuccessful as during the etching process, the wood oozed oily extractives. Most dense tropical wood species have high extractive content [46], but some dense temperate species, such as hornbeam (*Carpinus betulus* L.), have low extractive contents [47,48], and greater success in etching fine microstructural features might be achieved with such species. 

The most unexpected finding arising from this study was the extent to which high-energy plasma etching of basswood increased the prominence of rays at radial surfaces. Rays in unmodified basswood are discernable as short dark ribbons or flecks at radial surfaces [49]. Ray fleck in basswood is modest compared to that found in woods with much wider rays, such as oaks (*Quercus* spp.), sycamore (*Acer pseudoplatanus* L.), and some tropical species (for example, lacewood *Panopsis* spp. and leopard wood *Roupala montana* Aubl.) [49]. Ray fleck is regarded as an attractive aesthetic feature [50], and processes have been developed to enhance ray flecks at quarter-sawn wood surfaces. For example, ray fleck can be enhanced by exposing wood to gaseous ammonia (ammonia fuming), according to Peipher (2010) cited by Miklečić et al. [51]. Staining wood with solvent-soluble dye also enhances ray fleck because rays absorb less dye than fibrous tissues [52]. These methods of enhancing ray fleck find applications in the manufacture of furniture and cabinets rather than the construction industry. They are simpler and easier methods of enhancing ray fleck than the plasma etching process used here. However, it might be possible to develop and optimize plasma etching processes that could be more suitable for practical applications. Significant further research and development would be needed to create such processes.

## 5. Conclusions

It is concluded that: (1) The etching of radial longitudinal basswood surfaces is more uneven at radial versus tangential surfaces because of the physical and chemical heterogeneity of wood tissues that are present at radial surfaces; (2) the greater etch non-uniformity at radial basswood surfaces is due in part to the presence of ribbons of ray tissue (ray fleck) which were more resistant to plasma etching than ‘fibrous tissues. The implications of the findings (above) for the development of processes to etch microstructural features at wood surfaces and to enhance ray fleck at radial surfaces were discussed, and it was concluded that further research and development would be needed to create such processes. 

## Data Availability

The data presented in this paper are available on request from the corresponding author.
